# Protection from Intracellular Oxidative Stress by Cytoglobin in Normal and Cancerous Oesophageal Cells

**DOI:** 10.1371/journal.pone.0030587

**Published:** 2012-02-16

**Authors:** Fiona E. McRonald, Janet M. Risk, Nikolas J. Hodges

**Affiliations:** 1 School of Biosciences, The University of Birmingham, Edgbaston, Birmingham, United Kingdom; 2 School of Dentistry, The University of Liverpool, Liverpool, United Kingdom; Roswell Park Cancer Institute, United States of America

## Abstract

Cytoglobin is an intracellular globin of unknown function that is expressed mostly in cells of a myofibroblast lineage. Possible functions of cytoglobin include buffering of intracellular oxygen and detoxification of reactive oxygen species. Previous work in our laboratory has demonstrated that cytoglobin affords protection from oxidant-induced DNA damage when over expressed *in vitro*, but the importance of this in more physiologically relevant models of disease is unknown. Cytoglobin is a candidate for the tylosis with oesophageal cancer gene, and its expression is strongly down-regulated in non-cancerous oesophageal biopsies from patients with TOC compared with normal biopsies. Therefore, oesophageal cells provide an ideal experimental model to test our hypothesis that downregulation of cytoglobin expression sensitises cells to the damaging effects of reactive oxygen species, particularly oxidative DNA damage, and that this could potentially contribute to the TOC phenotype. In the current study, we tested this hypothesis by manipulating cytoglobin expression in both normal and oesophageal cancer cell lines, which have normal physiological and no expression of cytoglobin respectively. Our results show that, in agreement with previous findings, over expression of cytoglobin in cancer cell lines afforded protection from chemically-induced oxidative stress but this was only observed at non-physiological concentrations of cytoglobin. In addition, down regulation of cytoglobin in normal oesophageal cells had no effect on their sensitivity to oxidative stress as assessed by a number of end points. We therefore conclude that normal physiological concentrations of cytoglobin do not offer cytoprotection from reactive oxygen species, at least in the current experimental model.

## Introduction

Cytoglobin is a member of the globin family of haemoproteins that include haemoglobin and myoglobin, as well as the more recently identified neuroglobin that is expressed mainly in cells of the CNS [1]. The crystal structure of cytoglobin has been solved and studied in detail [2], [3], [4] showing that cytoglobin has many similarities to other globins, including the classic three-over-three alpha helical globin fold and a P_O2_ (oxygen affinity) of approximately 0.2 Torr, similar to that of myoglobin [e.g. 5], [6]. The high affinity of cytoglobin for oxygen has led to the suggestion that cytoglobin may serve as an “intracellular” oxygen transport system. In this scenario, cytoglobin is proposed to deliver oxygen to the mitochondria to sustain oxidative phosphorylation, in a manner similar to the function of myoglobin in muscle cells. Interestingly, oxygen affinity appears to be redox-sensitive, and regulated by the formation of a disulphide bridge between two external cysteine residues (Cys B2 and Cys E9). The redox-sensitive nature of cytoglobin oxygen affinity suggests a possible role of cytoglobin as an oxygen “sink/reserve”, whereby oxygen is only released when cells become hypoxic. Although these are attractive hypotheses, cytoglobin expression appears to be largely limited to cells of a fibroblast origin with no apparent correlation between metabolic activity of tissues and levels of cytoglobin expression which, in any case, is rather low in most cell types investigated [7], [8]. These findings have led to the search for alternative physiological function(s) for cytoglobin.

Cytoglobin was first identified in 2001 [9] during a proteomic screen of hepatic stellate cells isolated from fibrotic rat liver tissue and indeed was originally named stellate cell activation association protein (STAP) in recognition of that fact [9]. Subsequent work – both *in vitro* and *in vivo* [10]–[13], most recently using transgenic animals over-expressing cytoglobin [14], [15] appear to confirm that cytoglobin plays a role in the fibrotic response in a number of organs including the liver and kidney. Although the precise role of cytoglobin in fibrosis remains to be established, pertubation of redox homeostasis is a well characterised feature of fibrosis and there is good evidence (particularly with regards lung and kidney) that progression of fibrotic lesions involves cycles of oxidative reperfusion injury subsequent to tissue hypoxia. Furthermore, it has been demonstrated that cytoglobin expression can be upregulated by hypoxia [16]–[19]. Therefore it seems likely that cytoglobin is involved in the adaptive response associated with this injury.

Related to the findings in fibrotic disease, there is also an emerging body of mechanistic evidence to suggest that cytoglobin may afford protection from oxidative cellular injury under other circumstances, possibly as a result of its ability to scavenge reactive oxygen species [20]–[23]. Indeed, work in our own laboratory has shown that overexpression of cytoglobin reduces levels of chemically-induced reactive oxygen species (ROS) and affords protection from pro-oxidant induced injury (lipid peroxidation and oxidative DNA strand breaks) [24]. In further support for a role in detoxification of ROS, it has been reported that cytoglobin has peroxidase activity [9]. Interestingly, more recently it has been reported that ferric cytoglobin has pseudo-peroxidase activity against lipids, suggesting that, under conditions of oxidative stress, turnover of lipid based cell signalling molecules may also be part of a cytoglobin-dependent antioxidant response [25]. Furthermore, another vertebrate globin, globin X, has been found to be membrane associated and possibly involved in protection of lipids from oxidation [26].

Intriguingly, in addition to the possible roles discussed above, the cytoglobin gene (*CYGB*) is also a candidate tumour suppressor gene [27], [28], [29]and we have identified *CYGB* as a candidate tylosis with oesophageal cancer (*TOC*) gene [30]–[33]. Although *CYGB* is not mutated in tylotic individuals [31] or in a series of sporadic squamous cell oesophageal carcinomas [34], we have shown that its expression is strongly downregulated in non-cancerous oesophageal biopsies from patients with TOC compared with normal biopsies [33]. *CYGB* is also methylated and downregulated in sporadic oesophageal, lung and head and neck cancers [27], [28], [33]. Recently, downregulation of cytoglobin has also been shown to promote chemically induced carcinogenesis in rodent liver [35].

There is compelling evidence that oxidative damage to DNA (e.g. 8-oxo deoxyguanosine), if not repaired, contributes to the formation of mutations that are known to be important in the aetiology of human carcinogenesis [36], [37]. We therefore hypothesise that loss of cytoglobin expression would render oesophageal cells more susceptible to the damaging effects of ROS, and that this contributes to the observed phenotype of *TOC*. In the current study we tested this hypothesis by manipulating cytoglobin expression in both normal oesophageal epithelial cells and oesophageal squamous cell carcinoma cells, which have normal physiological and no endogenous expression of cytoglobin, respectively.

## Results

### Manipulation of cytoglobin expression

**Figure 1:**
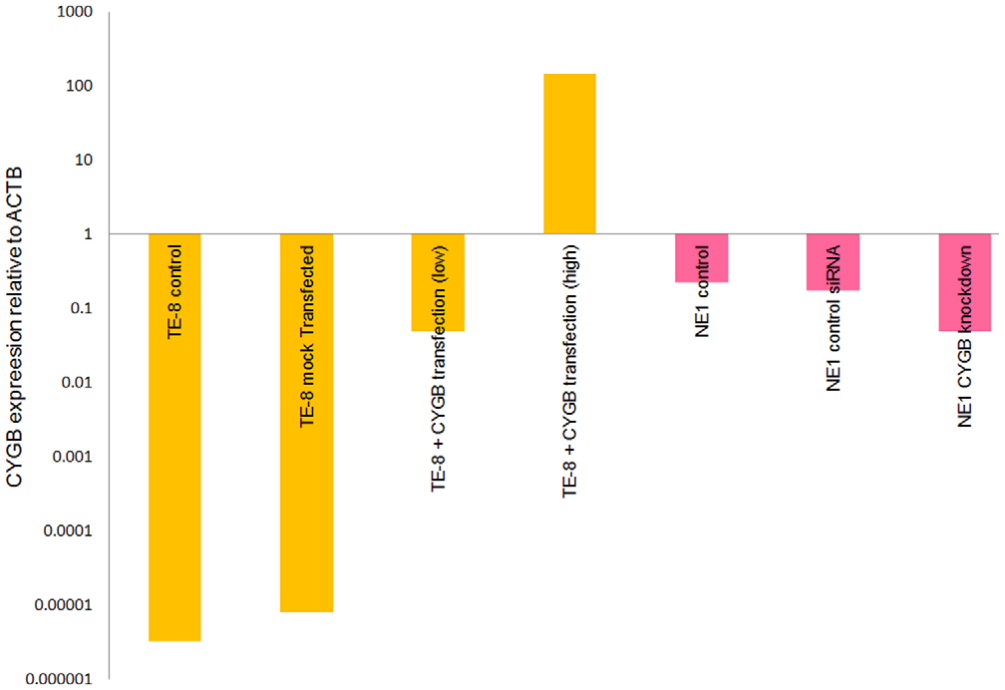
Quantification of CYGB expression in oesophageal cells subjected to transient transfection or siRNA knockdown. Levels of CYGB expression, compared to ACTB expression, were determined by real time RT-PCR in a parallel sample for each comet assay performed. These expression levels therefore relate directly to the comet assay data presented in Figure 3. The geometric mean of all experiments is shown for both TE-8 cells (orange bars) and NE1 cells (pink bars).

Real time RT-PCR showed that the TE-8 squamous cell oesophageal cancer cell line has extremely low endogenous levels of CYGB expression (lower than 10^−5^ with respect to beta actin (ACTB)), whereas NE1 normal oesophageal keratinocytes express CYGB at approximately 0.1× ACTB. This natural level of CYGB expression in NE1 cells was deemed to be the normal physiological level, as it is within the same order of magnitude as that observed in a panel of normal tissues (data not shown). CYGB expression was modulated in these two cell lines by either transient transfection of the gene (TE-8 cells) or transient knockdown using RNA interference (NE1 cells). Cytoglobin expression was artificially induced by approximately 4 or 7 orders of magnitude in TE-8 cells, and was reduced by approximately five-fold by siRNA knockdown in NE1 cells (Figure 1).

### Characterisation of cells

Normal oesophageal epithelial (NE-1) and oesophageal cancer (TE-8) cells were assayed for levels of oxidative stress using the redox-sensitive compound 2′,7′-dichlorodihydrofluorescein diacetate (H2DCF-DA) in the presence and absence of buthionine sulfoxamine (BSO).

**Figure 2:**
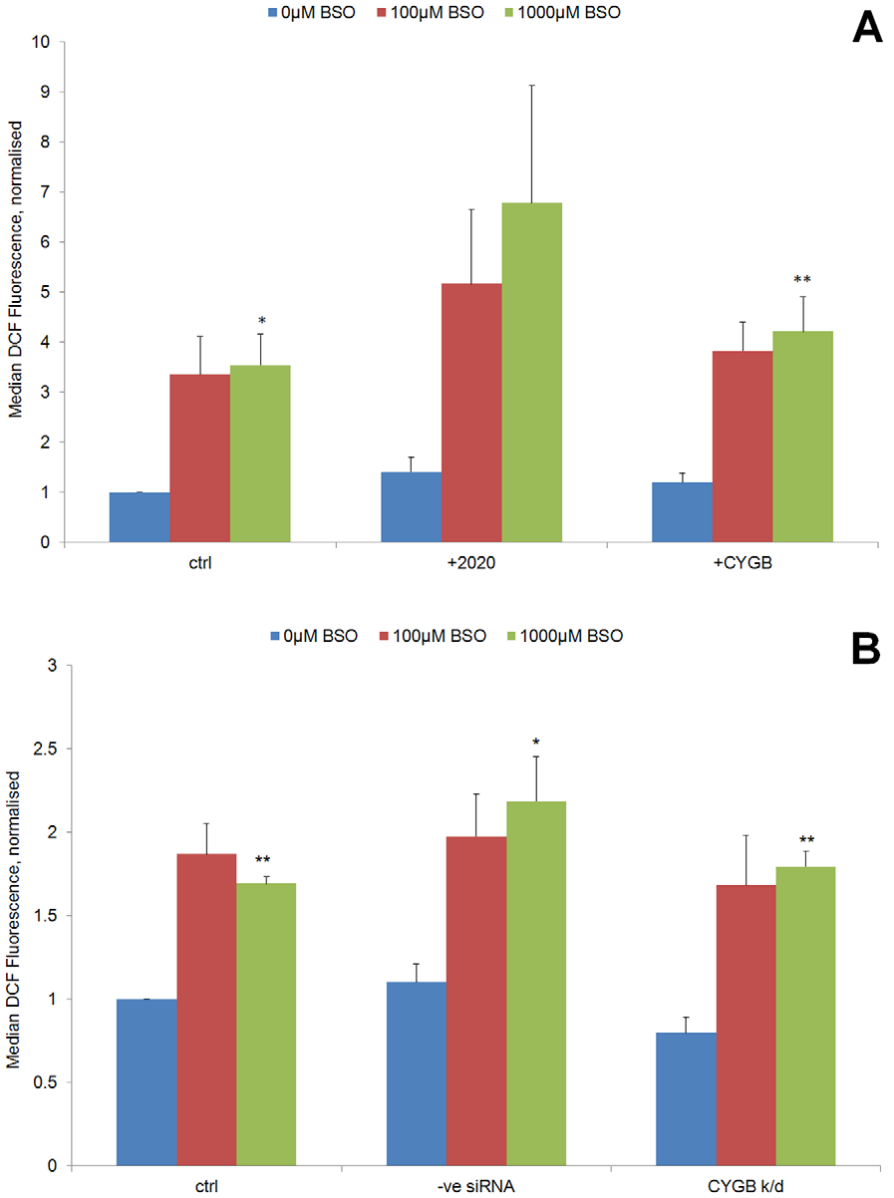
Impact of BSO treatment and CYGB expression on oxidative stress response. Oxidative stress was measured in a: TE-8 and b: NE1 cells. Median DCF fluorescence is the mean of 5 repeats. All values were normalised to the level seen in the control cells with no BSO. Error bars represent SEM.+2020: transfection reagent only; +CYGB: cells transfected with full length CYGB; −ve siRNA: scrambled control; CYGB k/d: CYGB siRNA. * Statistically significant concentration-dependent response (p<0.05) ** Statistically significant concentration-dependent response (p<0.01).

The results indicated that BSO was able to induce oxidative stress in both TE-8 and NE1 cell lines, with a concentration-dependent response being evident (Figure 2). In TE-8 cells, this reached statistical significance in unmanipulated cells (p = 0.0134) and CYGB transfected cells (p = 0.0034), but not in mock transfected cells (p = 0.092). Nevertheless, a similar trend was observed in mock transfected cells. In the normal oesophageal cell line NE1, similar results were obtained: in this instance the BSO concentration-dependent oxidative stress response was statistically significant in all three groups of cells. BSO was able to induce oxidative stress in control cells (p = 0.0032), negative control siRNA treated cells (p = 0.0283), and cells subjected to siRNA knockdown of CYGB (p = 0.0077). Although a trend was observed in TE-8 cells towards overexpression of CYGB being associated with lower levels of ROS when compared with mock transfected cells, this was not statistically significant. No effect of CYGB knockdown was observed in NE1 cells. However, as our primary question was to look at ROS-associated DNA damage, and not oxidative stress *per se*, we next performed the comet assay on the two cell lines: this has the additional benefit of being a much more sensitive indicator of cellular oxidative damage.

### Relationship between oxidative DNA damage and cytoglobin expression

#### Comet Assay

**Figure 3:**
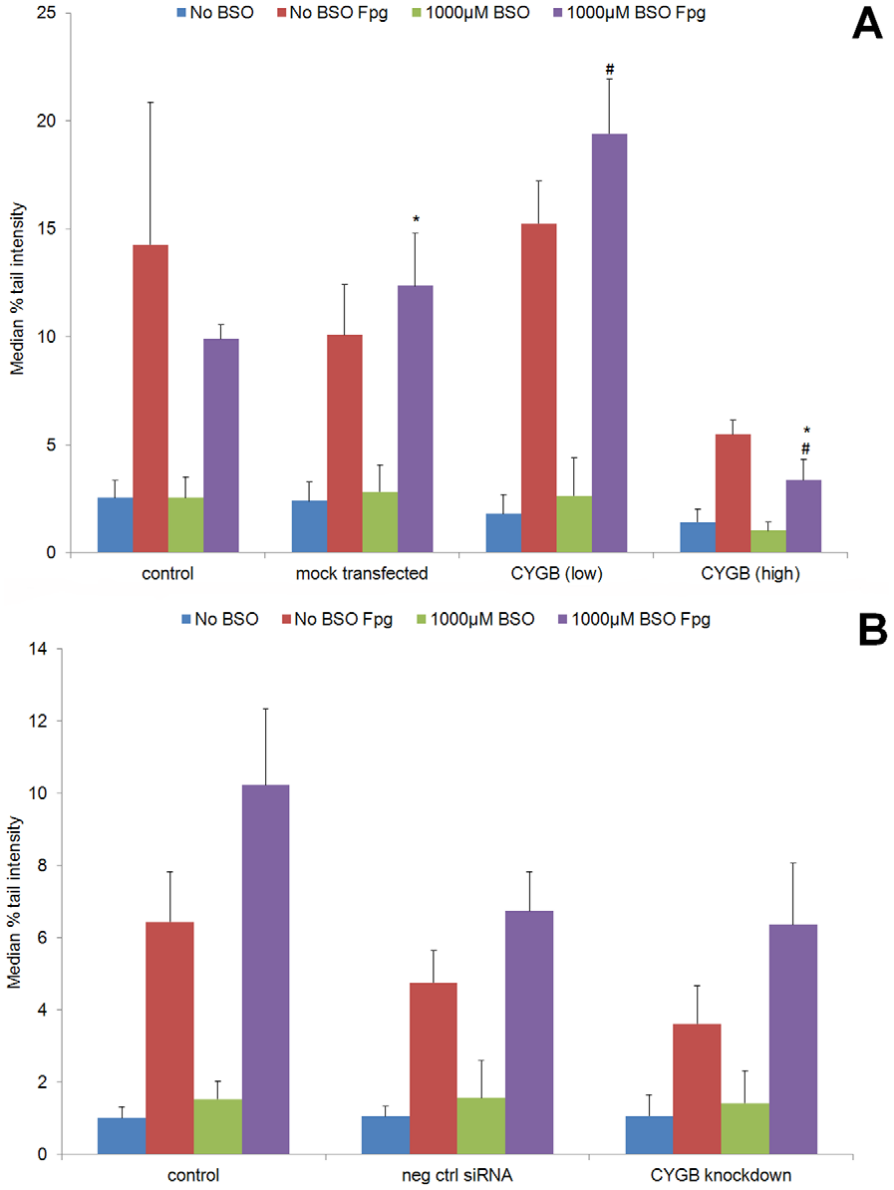
Impact of BSO treatment and CYGB expression upon Oxidative DNA damage. DNA strand-breaks (baseline and oxidative-damage-induced) were measured by the Fpg-modified comet assay in a: TE-8 and b: NE-1 cells with different levels of CYGB expression (as shown in Figure 1). Mean of median percent comet tail intensity is shown. Error bars represent SEM. * Significantly different from each other (p<0.05). # Significantly different from each other (p<0.05).

In TE-8 cells, CYGB expression was artificially restored by transient transfection (Figure 1). Oxidative DNA damage was measured by the comet assay in cells with two different levels of CYGB expression in addition to the baseline controls: artificially high overexpression (approximately 10^2^–fold with respect to ACTB expression), and low (roughly physiological) expression (approximately 10^−1^–fold with respect to ACTB expression). At artificially high levels of CYGB expression, a significant reduction in oxidative DNA damage was observed under conditions of exogenous oxidative stress (1000 µM BSO) (p = 0.014) when compared to mock-transfected cells (Figure 3a) There was also a significant difference in oxidative DNA damage between cells with low/physiological and high CYGB expression levels (p = 0.035). However, there was no significant difference between mock-transfected cells and those with low/physiological CYGB expression levels (p = 0.074); similarly no difference was observed between control and mock-transfected cells (p = 0.455). These data suggest that CYGB exerts a protective effect against cellular oxidative damage only at artificially high – non-physiological levels of expression. Although, counter-intuitively, there was a trend towards increased oxidative DNA damage when CYGB was expressed at low/physiological levels, this trend did not reach statistical significance. In NE1 cells, reduction of CYGB expression by 90% using RNA interference did not result in any difference in oxidative DNA damage: this further supports the argument that CYGB only exerts its cytoprotective effects at artificially high (non-physiological) levels of expression (figure 3b).

### Cytoglobin and cell motility

Previous studies have shown that cytoglobin influences cellular motility and colony forming ability [29], however in the current study no effect of cytoglobin knockdown upon cell growth was detected in either NE1 oesophageal keratinocytes or CCD-18Co colonic myofibroblasts, at either 25 or 50 hours post-wounding (data not shown).

## Discussion

Previous work in our and other laboratories has shown that cytoglobin has the potential to detoxify reactive oxygen species (ROS) *in vitro* when overexpressed in various cell culture systems [20], [21], [22], [24]. Furthermore, downregulation of cytoglobin by RNAi has also recently been shown to sensitise glioma cells to oxidative stress, induced by both inhibition of the electron transport chain with antimycin A, and ionizing radiation [23]. Further support for a role for cytoglobin in detoxification of ROS is the biochemical evidence that the cytoglobin protein possesses intrinsic peroxidase activity as well as antioxidant properties and there is evidence that other globins including neuroglobin and globin X may also have similar properties [20], [26], [38], [39]. However, in the case of cytoglobin, it remains uncertain whether this is a genuine physiological function of cytoglobin or an artifact of the experimental models used. To date, no “cytoglobin reductase” has been identified that could reduce the Fe^3+^ back to Fe^2+^, so it is not clear how oxidised cytoglobin would be recycled within cells. There is also evidence that cytoglobin has catalytic activity against the reactive nitrogen species nitric oxide, catalysing its conversion to nitrate where ascorbate and cytochrome b(5) have been identified as potential sources of reducing power [40]. This putative activity correlates well with the observation of high levels of cytoglobin expression in fibroblasts and smooth muscle cells of the vasculature, where nitric oxide is a well-known signalling molecular that mediates vascular tone [41]. However, other studies have questioned whether nitric oxide dioxygenase is a physiologically relevant enzymic activity of cytoglobin [42]. In addition, there is also emerging *in vivo* evidence of a protective role of cytoglobin (and neuroglobin) against oxidative stress, especially in models of fibrosis that involve hypoxic reperfusion and subsequent oxidative injury [10]–[15].

Much of these data have been generated in experimental models where cytoglobin is over-expressed, so although these are interesting findings, their physiological relevance remains unclear. In the current study, we investigated whether cytoglobin expression could afford protection from chemically-induced oxidative DNA damage in cultured oesophageal cells. This is particularly pertinent because *CYGB* is down regulated in both tylosis with oesophageal cancer and sporadic oesophageal cancers [33] and we therefore hypothesised that loss of cytoglobin expression would sensitise these cells to DNA damage and that this may be involved in the aetiology of oesophageal cancer. When cytoglobin was overexpressed in TE-8 oesophageal cancer cell lines, which have little or no endogenous cytoglobin expression, consistent with previous studies we observed statistically significant protection from BSO-induced oxidative DNA damage as assessed by the FPG-modified comet assay. This effect however was concentration-dependent: at lower levels of cytoglobin overexpression the protective effect was lost. Consistent with this, knockdown of physiological levels of cytoglobin in cultures of primary NE1 oesophageal cells did not sensitise them to BSO-induced oxidative DNA damage as assessed by the same endpoint. Interestingly, it has recently been reported that knockdown of cytoglobin expression in glioma cells by RNAi elevates levels of antimycin A-induced hydrogen peroxide [23]. In the current study, we observed no significant difference in levels of BSO-induced oxidative stress as assessed by oxidation of dichlorofluoroscein. The reason for this difference is not clear but may be related to either the different cell line or method to induce oxidative stress.

Our findings suggest that although induction of cytoglobin expression,for example as a result of hypoxia or in response to oxidative injury, has the potential to be protective, in our study this was only observed at CYGB levels unlikely to be achieved *in vivo*. Furthermore, there was no evidence of an increased sensitivity to ROS following knockdown by RNAi in cells expressing cytoglobin at physiological levels. Therefore, a cytoprotective role against oxidatively induced DNA damage does not appear to be a function of cytoglobin in oesophageal cells and other explanations of cytoglobin function and loss of expression in this disease model are required.

## Materials and Methods

### Cell culture

Normal oesophageal epithelial cells (NE-1, A gift of Professor G.S.W Tsao, Department of Anatomy, University of Hong Kong (see [43]) were maintained in T_75_ culture flasks using keratinocyte serum free media (Gibco) supplemented with bovine pituitary extract (25 µg ml^−1^) and recombinant epidermal growth factor (0.15 ng ml^−1^). Cultures were routinely split 1∶3 approximately every 3–4 days using trypsin-EDTA and soybean trypsin inhibitor (Gibco) according to the recommended protocol for the keratinocyte serum-free medium. The oesophageal cancer cell line TE-8 (A gift of Professor Toshio Kudo, Cell Resource Centre for Biomedical Research, Tohoku University, Japan, [44]) was grown in T_75_ culture flasks with RPMI medium supplemented with 10% FBS and 2% L-Glutamine. TE-8 cells were split as necessary using a standard trypsin-EDTA protocol. CCD-18Co colonic myofibroblasts (purchased from ATCC product no: CRL-1459; [45]) were grown in DMEM supplememented with 10% FBS, 1% L-glutamine and 1% non-essential amino acids (NEAAs). These cells were spilt as necessary using a standard trypsin-EDTA protocol.

### Cytoglobin knockdown

NE-1 cells or CCD-18Co cells were subcultured into six-well plates (for comet assay) or twelve-well plates (for ROS assay and wound-healing assay), and allowed to reach 60–70% confluence prior to transfection. Cells were transfected with 25 nM final concentration of either a negative control scrambled siRNA (Ambion; AM4611) or CYGB siRNA (Qiagen; SI03228323 or Ambion; s41571) using 15 µg ml^−1^ TransIT siQuest reagent (Mirus Bio) according to the manufacturer's instructions. Following RNAi knockdown, cells were incubated for 42 hours before carrying out experiments (24 hours for wound-healing experiment). Expression of CYGB was assayed by RT-PCR (see below).

### Overexpression of cytoglobin

TE-8 oesophageal squamous cell carcinoma cells were subcultured into six- or twelve-well plates as previously. Transient transfection of *CYGB* was accomplished with Transit 2020 transfection reagent (Mirus Bio) (2 µl or 5 µl per well for 12-well or 6-well plates respectively), and full length *CYGB* cDNA clone in a pCMV6-AC vector (Origene) (0.6 µg or 1.5 µg for 12-well or 6-well plates respectively), as per the standard protocol for the transfection reagent. Following transfection, cells were incubated for 42 hours before carrying out experiments. Expression of CYGB was assayed by RT-PCR (see below).

### RNA extraction, first strand cDNA synthesis and RT-PCR analysis

Isolation of total RNA was carried out using an RNeasy Minikit (Qiagen) with direct lysis of the cells in the culture plate, and homogenization using QiaShredder tubes. Reverse transcription was performed using a RETROscript Kit (Ambion) with, oligo dT primers and 1 µg of total RNA, according to the manufacturer's instructions. For RT-PCR a master mix was prepared containing TaqMan 2× master mix (Applied Biosystems), β-actin VIC/MGB probe for the endogenous control (Applied Biosystems 4326315E), CYGB primer and probe mix (sense 5′-CTC TAT GCC AAC TGC GAG GAC-3′, anti-sense 5′-AAC TGG CTG AAG TAC TGC TTG-3′ and probe FAM-5′-TGG CCA TCC TGG TGA GGT TCT TTG TG-3′-black hole quencher) in RNase-free water. A standard two-step protocol was employed for real time RT-PCR analysis (50°C for 2 minutes, followed by 50 cycles of 95°C for 15 minutes and 60°C for 1 minute). The Cycle Threshold (Ct) values obtained were used to calculate the real-time quantification value (RQ) utilising the ΔΔCt method, with ACTB Ct values and untreated/untransfected cells used as the normalisation factors.

### FPG modified comet assay

The method was based on that of Singh et al [46] with minor modifications [47]. Following treatment, cells were washed in cold PBS and gently scraped into 1 ml fresh PBS (TE-8 cells) or trypsinised (NE1 cells). Following centrifugation (8000 rpm, 5 minutes) pellets were re-suspended in PBS (150 µl). An aliquot of re-suspended cells (30 µl) was placed into a sterile tube containing low melting point agarose (300 µl) and this cell suspension was transferred to two glass microscope slides (150 µl per slide), pre-coated with 0.5% normal melting point agarose. Glass coverslips were added and slides placed on a metal tray over ice for 10 minutes. Coverslips were removed and slides incubated for 1 h at 4°C in lysis buffer (2.5 M NaCl, 0.1 M Na_2_EDTA, 10 mM Tris base, 1% sodium N - lauryl sarcosinate, 10% DMSO and 1% Triton X-100). Slides were washed (3×5 minutes) with FPG Buffer (40 mM HEPES, 0.1 M KCl, 0.5 mM EDTA 0.2 mg/ml bovine serum albumin pH 8.0) and incubated with 1 unit of FPG enzyme (Trevigen) in 50 µl FPG buffer at 37°C for 1 hour (control slides were incubated with FPG buffer only). Slides were transferred to a horizontal electrophoresis tank containing electrophoresis buffer (300 mM NaOH and 1 mM Na_2_EDTA, pH 13.0) and DNA allowed to unwind for 20 minutes. DNA was subjected to electrophoresis (25 V, 0.8 V cm^−1^, 20 minutes) and slides neutralised by washing (3×5 minutes) with neutralisation buffer (0.4 M Tris, adjusted to pH 7.5). Slides were subsequently stained with Sybr Gold 50 µl (Invitrogen, 10× solution in neutralisation buffer).

The slides were examined at 320× magnification using a fluorescence microscope (Zeiss Axiovert 10, Germany), fitted with a 515–560 nm excitation filter and a barrier filter of 590 nm. A USB digital camera (Merlin, Allied Vision Technologies) received the images, which were analysed using a personal computer-based image analysis system Comet assay IV (Perceptive instruments). Images of one hundred randomly selected nuclei were analysed per slide. Measurement of percent tail DNA (TD %) was chosen to assess the extent of DNA damage as this has been shown to suffer much less from inter-run variation than other comet parameters because it is largely independent of electrophoresis voltage and run time [48]. Median values of three separate experiments were analysed using ANOVA and post-hoc Student's t-test, as recommended by Duez et al [49].

### ROS assay

Reactive oxygen species were assessed by measuring the oxidation of the redox sensitive dye 2′,7′-dichlorodihydrofluorescein diacetate (H2DCF-DA). Following hydrolysis by intracellular esterases, the resultant H2DCF is unable to leave the cell and is then oxidized by ROS to form the fluorescent DCF molecule. The level of fluorescence is proportional to the degree of oxidative stress in the cells. In order to induce oxidative stress artificially, buthionine sulfoximine (BSO), which inhibits the rate-limiting step in glutathione synthesis, was added to the cultured cells for 24 hours prior to the experiment at 100 or 1000 µM.

Briefly, following treatments 2′,7′-dichlorodihydrofluorescein diacetate (final concentration 10 µM for TE-8 cells; 1 µM or 0.1 µM for NE1 cells) was added and cells incubated at 37°C for 30 minutes in the dark. Cells were detached by trypsinisation, pelleted by centrifugation and resuspended in PBS. Sample fluorescence intensity was analysed (FITC channel) using a FACScalibur™ flow cytometry (Becton Dickinson) and CellQuestTM Pro software (Becton Dickinson). Cells without fluorescent dye were used as a negative control to correct for background autofluorescence.

### Wound Healing Assay

NE1 oesophageal keratinocyte cells and CCD-18Co colonic myofibroblasts were grown in twelve-well plates, and subjected to RNAi knockdown of CYGB as previously described, using both Ambion and Qiagen siRNAs to CYGB, and a scrambled control siRNA. Each condition was replicated six times for each cell line. The cell layer was scratched in a cross shape using a sterile pipette tip at 24 hours post-knockdown. Cells were photographed at 1, 25 and 50 hours post-wounding using a USB digital camera. Images were analysed using TScratch software [50], which analysed alterations in the percentage of Open Wound Area (OWA).
